# Pathophysiology and clinical aspects of epiretinal membrane – review

**DOI:** 10.3389/fmed.2023.1121270

**Published:** 2023-08-10

**Authors:** Mateusz Kamil Ożóg, Marta Nowak-Wąs, Wojciech Rokicki

**Affiliations:** ^1^Department of Histology and Cell Pathology, Faculty of Medical Sciences in Zabrze, Medical University of Silesia, Katowice, Poland; ^2^Department of Histology, Cytophysiology and Embryology, Faculty of Medicine, Academy of Silesia, Zabrze, Poland; ^3^Department of Ophthalmology, Kornel Gibiński University Clinical Center, Medical University of Silesia, Katowice, Poland; ^4^Department of Ophthalmology, Faculty of Medical Sciences in Katowice, Medical University of Silesia, Katowice, Poland

**Keywords:** epiretinal membrane, macular pucker, myofibroblasts, pre-macular fibrosis, retina, cellophane maculopathy

## Abstract

The epiretinal membrane (ERM) is a pathological tissue formed at the vitreoretinal interface. The formation of this tissue is associated with numerous symptoms related to disturbances of vision. These types of lesions may arise idiopathically or be secondary to eye diseases, injuries and retinal surgeries. ERM tissue contains numerous cell types and numerous cytokines, which participate in its formation. The aim of this paper is to summarize information about the etiology, epidemiology, pathophysiology and treatment of ERM, with a brief description of the main cells that build the ERM – as well as the cytokines and molecules related to ERM pathogenesis – being provided in addition.

## 1. Introduction

The epiretinal membrane (ERM), commonly known as macular pucker or cellophane maculopathy, is a pathological tissue formed at the junction of the vitreous body and the retina – the vitreoretinal interface. The formation of this tissue is associated with symptoms related to visual disturbances. The lesions of this type may arise idiopathically or be secondary to eye diseases, injuries and retinal surgery. In the case of idiopathic changes, a number of factors may favor the development of these lesions (1).

ERM tissue contains many cell types deriving from different parts of eyeball: retinal pigment epithelium (RPE) cells, fibrocytes, fibrous astrocytes, myofibroblast-like cells, glial cells, endothelial cells (ECs), and macrophages. The cellular composition of this tissue varies individually and depends on the cause of the lesions and the participation of numerous cytokines such as growth factors, tumor necrosis factor (TGF) or chemokines (2).

The aim of this paper is to summarize information on the etiology, epidemiology, pathophysiology, and treatment of ERM.

## 2. Pathophysiology of ERM

The vitreous body, consisting of a colorless, highly hydrated gel matrix, fills the space called the vitreous chamber located posteriorly to the ciliary body. The vitreous body adheres loosely to the retina, connecting most strongly around the ora serrata and the optic nerve (3). Its outer layer – vitreous cortex – is made of collagen, while the inside is filled with vitreous humor containing mainly water (98–99%), fibrous protein called vitrosin, type II collagen fibers, glycosaminoglycans, hyaluronates, opticins and other proteins (4).

The vitreous body contains a small number of cells, mainly phagocytes, that remove cellular debris and hyalocytes the main function of which is to produce hyaluronans (5). Vitreous body is involved in maintaining proper intraocular pressure, protecting the lens against oxidative stress and is one of the optical centers (3, 6).

**Figure 1:**
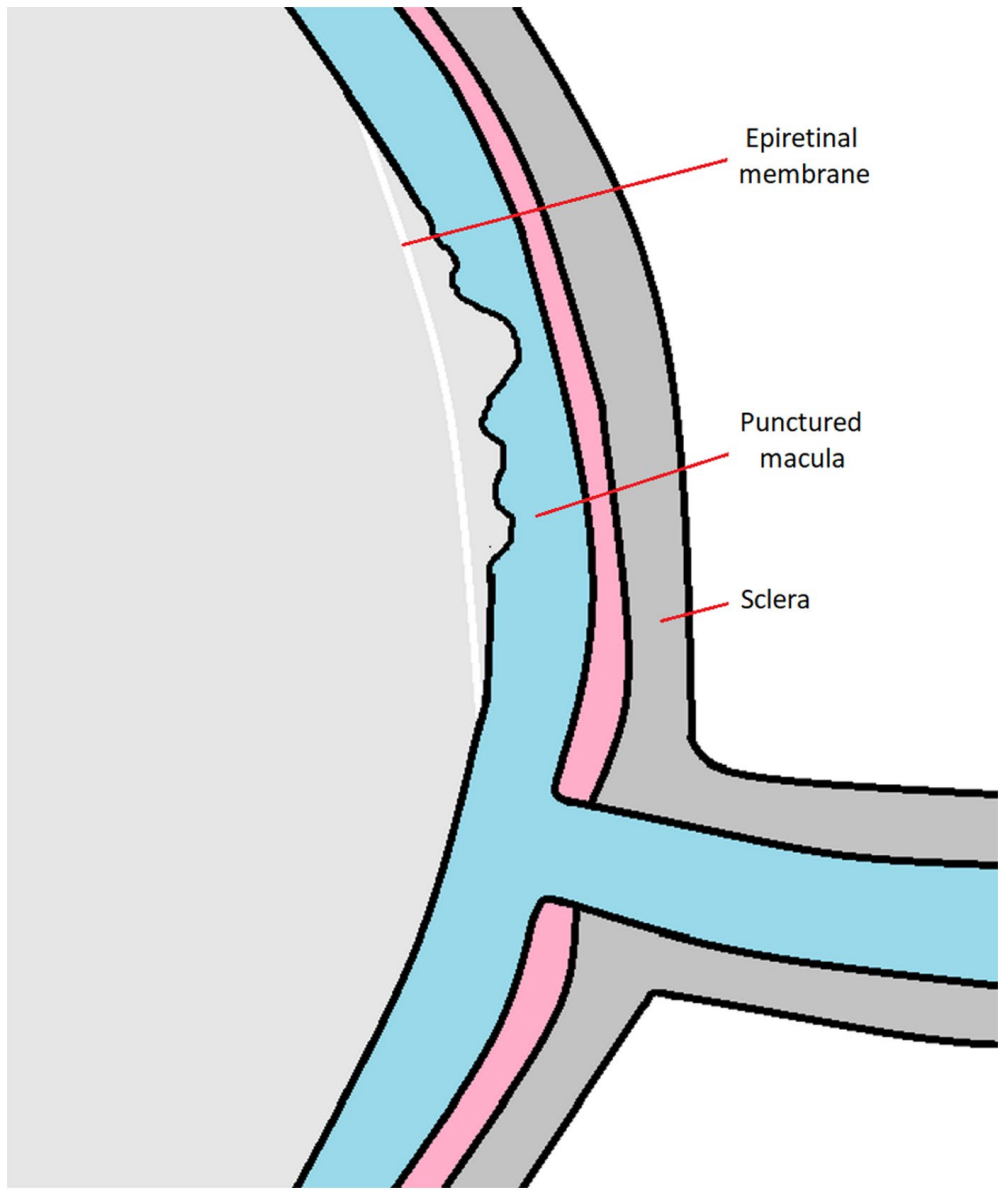
This illustration presents ERM tissue in relation to other eyeball parts.

With age, an increase in liquefaction and fiber aggregation occurs, one which may lead to many ophtalmic diseases (7, 8). As a result of these processes, the volume of the vitreous body decreases, the said body collapses, and the collagen fibers reorganize. This leads to changes in the shape of the vitreous body and posterior vitreous detachment (PVD). If this process is not complete, and macula and vitreous body come into contact, a posterior vitreomacular adhesion (VMA) is formed at the point of this contact. This process may lead to the formation of vitreomacular traction (VMT), which involves foveal contour distortion and retinal layer disorders, with a possible elevation of the retina above the pigment epithelium (RPE), not interrupting, however, the retina’s continuity. In this state most patients self-heal due to completion of PVD. When VMT is not self-healed and a hole in the macula occurs, the next step in the evolution of the pathology could be vitreoschisis, which occurs in the case of the half of PVD patients. During this process posterior vitreous cortex splits, leaving the outermost layer attached to the macula, while the remainder of the vitreous collapses forward. This can lead to the proliferation within the retinal vitreous residue, i.e., the formation of an epiretinal membrane (ERM) (9, 10) (Figure 1).

**Figure 2:**
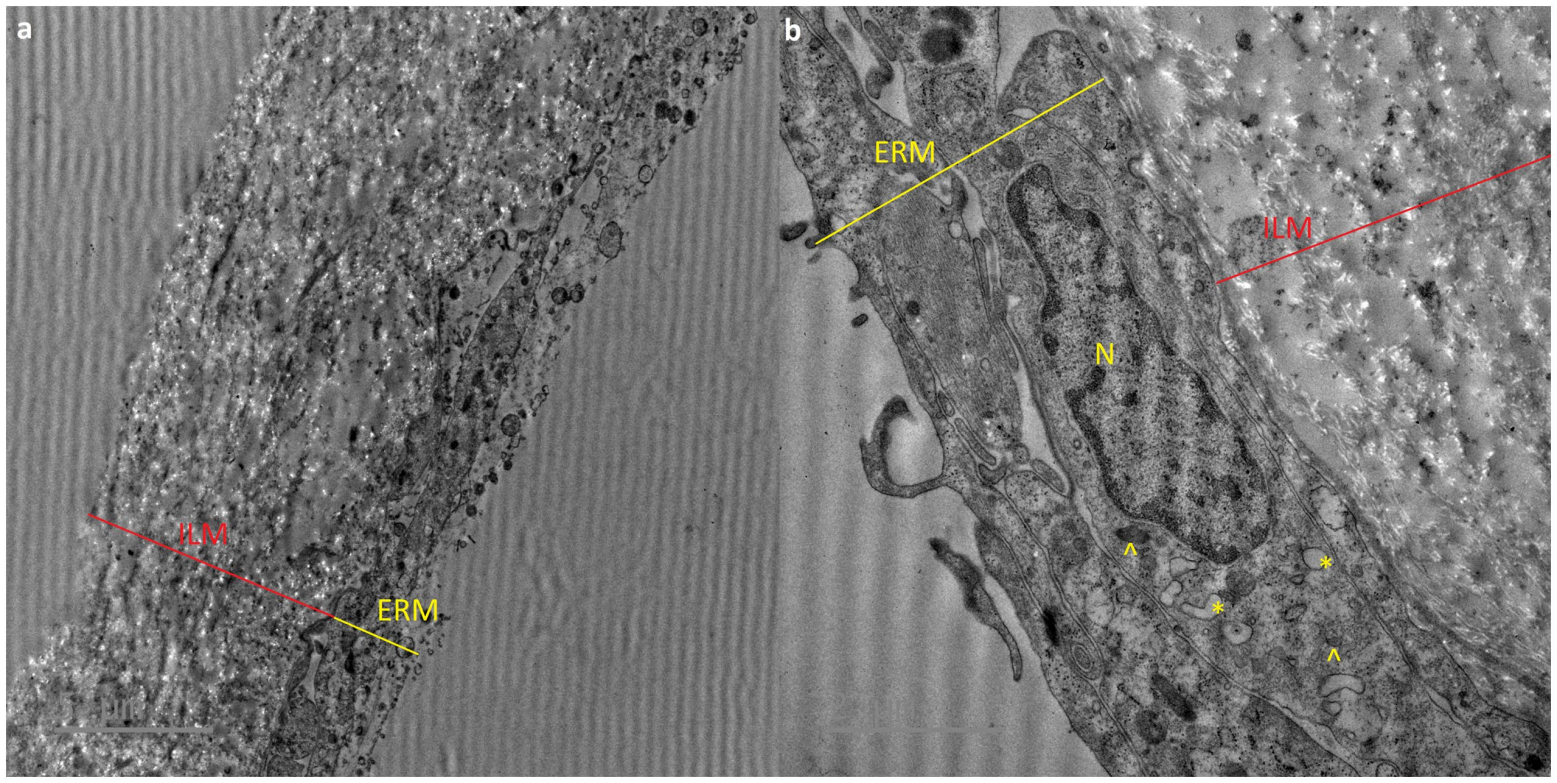
Transmission electron micrograph showing epiretinal membrane (ERM) and internal limiting membrane (ILM). a – original micrograph showing comparison of ERM and ILM structure (original magnification x4200), b – micrograph showing detailed structure of ERM and phagocytes: N – cell nuclei, asterix (*) – vacuoles, arrowhead (^) – dense granules (magnificated and focused micrograph a).

Formed ERM tissue can cause macular edema or distortion of the vitreoretinal interface, which may lead to visual disturbances, including blurred vision and a reduction of visual acuity (11) (Figure 2).

### 2.1. ERM tissue formation

**Figure 3:**
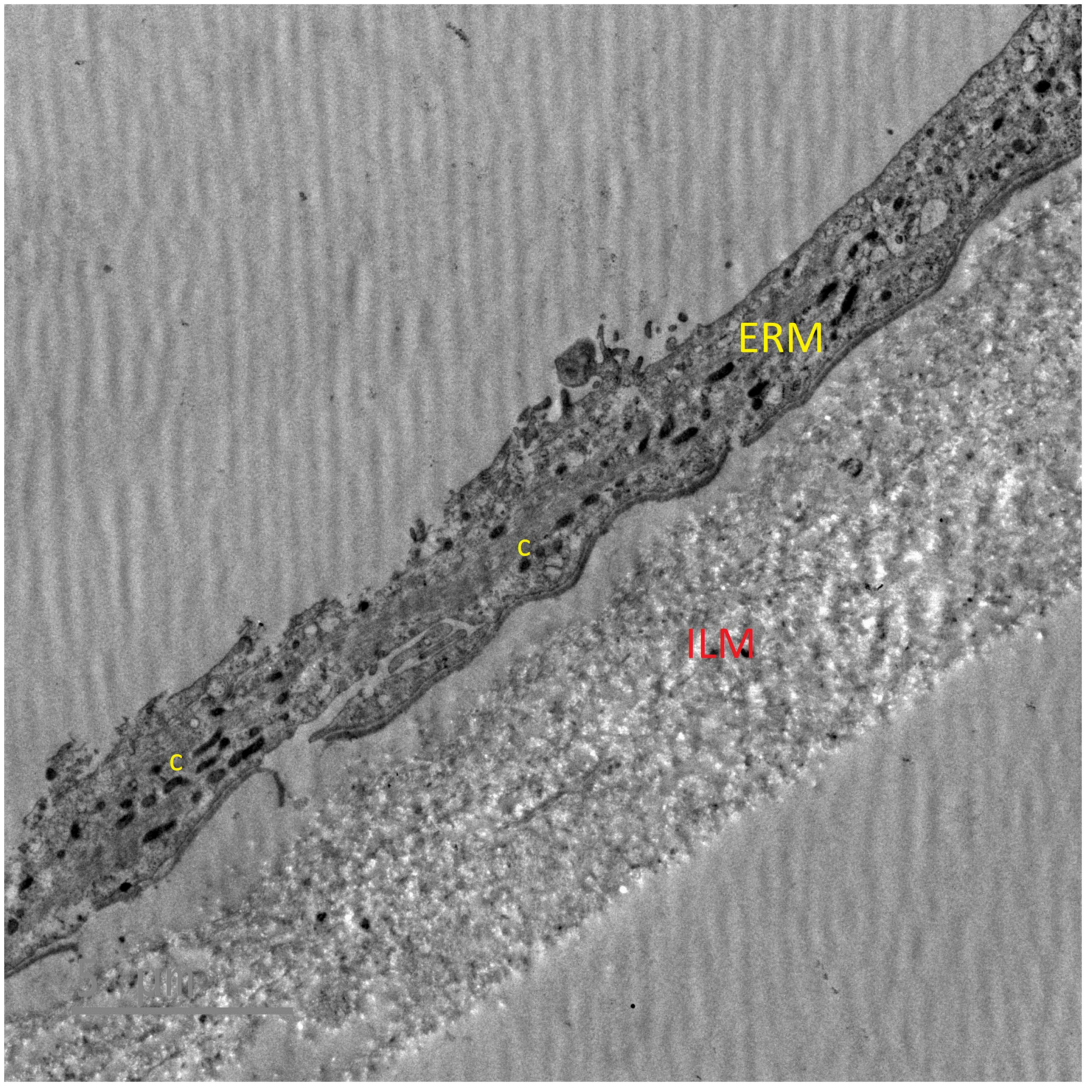
Transmission electron micrograph showing collagen fibers in epiretinal membrane. ERM – epiretinal membrane, ILM – internal limiting membrane, c – collagen fibers.

Pathological cell proliferation on the internal limiting membrane (ILM) surface is the basis for the formation of pathological tissue at the interface between the vitreous body and the retina. As a result of PVD, ILM dehiscence occurs, which leads to the migration of microglial cells to the surface. Microglial cells then interact with the neighboring hyalocytes and laminocytes of the vitreous cellular membrane (12). These cells then differentiate into fibroblast-like cells, which are directly responsible for the formation of the collagen scaffolding of the ERM (13, 14) (Figure 3).

ERMs can arise idiopathically or secondary to certain disease states, and as a result of a trauma or surgery within the eyeball.

In the case of the idiopathic ERM retinal glial cells, hyalocytes, fibroblasts, and myofibroblasts generated by cell migration and differentiation of microglial cells, hyalocytes, and laminocytes predominate (2, 14).

In secondary ERM, the presence of the retinal pigment epithelial cells, macrophages, T cells, and B cells is observed due to inflammation appearing in the etiology of this lesion (15, 16).

The leading theory presents the sequence of changes leading to the emergence of the ERM thus:

1. Microglial cells migrate to the surface of the retina as a consequence of PVD and the resulting formation of the cracks within the ILM.
2. The fragments of the vitreous membrane remaining on the surface of the ILM contain hyalocytes, which, due to contact with microglial cells, differentiate into myofibroblasts, while microglial cells may differentiate into fibroblasts.
3. PVD-induced ILM avulsion enhances the action of some ERM-promoting cytokines (17–19).

### 2.2. Idiopathic ERM

The idiopathic form of ERM affects approximately 95% of patients and is directly related to cellular proliferation resulting from PVD.

It is possible to distinguish 2 types of iERM. Type I is formed due to collagen of the vitreous body coming into contact with the internal limiting membrane (ILM) of the retina, which results in the production of a collagen membrane separating the two structures. Type II, on the other hand, results from cell proliferation that takes place directly on the ILM surface with or without a small layer of collagen in between (20).

### 2.3. Secondary ERM

The secondary form of ERM develops in the course of eye diseases or as a result of injuries of the eyeball or the surgeries performed on it.

As a result of these disorders, inflammation within the retina occurs, which leads to an inflammatory infiltration and, ultimately, a migration of cells from other layers of the retina to the ERM being in the process of formation (21) (Table 1).

**Table 1:** Diseases associated with secondary ERM.

Type of complication	Disease entities
Iatrogenic	Cataract surgery
	Vitrectomy surgery
	Retinopexy (laser or cryotherapy)
Retinal vascular diseases	Diabetic retinopathy
	Retinal vascular occlusive disease
	Coat’s disease
	Retinal arteriolar macroaneurysm
	Radiation retinopathy
	Sickle-cell retinopathy
Intraocular tumors	Retinal haemangioblastoma
	Vasoproliferative tumor
	Choroidal melanoma
	Hamartoma of the retina and retinal pigment epithelium combined
	Retinal astrocytic hamartoma
Vitreomacular traction disorders	Macular hole
	Vitreomacular traction syndrome
Other retinal diseases	Retinal tears
	Retinitis pigmentosa
Other diseases of the eyeball	Uveitis
	Myopia
	Trauma
	Age-related macular degeneration
	Neurofibromatosis type 2

## 3. Histopatology of ERM

### 3.1. The cells that build ERM

ERM is usually made up of two layers placed on the ILM. The outer layer directly overlying the ILM consists of randomly oriented proteins, while the inner layer consists of one or more layers of cells from the retina (22). The cells observed are glial cells, hyalocytes, RPE cells, macrophages, fibroblasts, and myofibroblast-like cells. Apart from the latter type, the source of the cells within the ERM is uncertain. It is known, however, that myofibroblast-like cells are formed by differentiating from other cell types within ERM (22, 23) (Table 2).

**Table 2:** Cells that built epiretinal membranes.

Type of cells	Description
Glial cells	Microglia are small cells of a monocytic / macrophagal origin, placed in retina and optic nerve. Those cells as parts of ERM tissue produce TGF-beta, which is mainly involved in the differentiation of myofibroblast-like cells (24).
	Within the ERM there is low abundance of astrocytes (which migrate from retina and optical nerve), the presence of which appears more prominent in other vitreomacular traction disorders such as vitreomacular traction syndrome (VMTS), lamellar macular holes (LMHs) and myopic traction maculopathies (24).
	The proliferation of Muller cells (a characteristic type of cells in retina) is believed to be one of the direct causes of ERM formation as source of proinflamatory cytokines (25, 26).
Hyalocytes	These are phagocytic cells, also of monocytic/macrophagal origin found on the surface of the vitreous base. Main function of this cells is production of elements of the extracellular matrix. These cells are responsible for the production of TGF-b which is a major contributor to the differentiation of myofibroblast-like cells (27, 28).
Retinal pigment epithelium (RPE) cells	These cells, originally forming single layer arranged at the outermost layer of the retina, migrate through retinal breaks and attach themselves to the inner retina. They are likely to transform to myofibroblast-like cells (22).
Macrophages	The presence of macrophages within lesion (which could be found by default in cornea, uveal tract and choroid) can be observed mainly in the secondary ERM associated with vitreous haemorrhage. They are the source of many cytokines involved in the pathophysiology of the lesion (29, 30).
Fibroblasts and myofibroblast-like-cells	These cells are responsible for the production of proteins forming the ERM intercellular matrix and produce many pro-inflammatory cytokines (22).

### 3.2. Cytokines and molecules related to ERM pathogenesis

A number of cytokines and molecules related to this process which directly or indirectly influence the development of this pathology have been identified. These cells are responsible for the production of proteins that constitute the ERM extracellular matrix (31) (Table 3).

**Table 3:** Cytokines and molecules involved in epiretinal formation.

Cytokine or molecule	Description
Vascular endothelial growth factor (VEGF)	VEGF is a growth factor that stimulates mitosis and endothelial cell migration and chemotactic factor for leukocytes. One of the main factors stimulating the production of VEGF is tissue hypoxia – in the case of its appearance, any type of cell is able to commence the production of VEGF (23).
	One of the phenomena observed in the course of PDR is capillary oclusion in the retina, which, by causing hypoxia, increases the expression of VEGF by the cells that build the retina (32). Pathological retinal angiogenesis promotes the formation of subretinal hemorrhages, which in turn promotes the development of ERM (33).
	Increased VEGF expression has been observed not only in the retina of ERM patients, but also within the lesion itself. Additionally, the presence of VEGF receptors in ERM-forming cells was demonstrated. This suggests that VEGF acts as a growth factor for ERM-forming cells (34, 35).
Placenta growth factor (PlGF)	PlGF is a homologue of VEGF that shares receptors with it, but displays significantly lower mitogenic properties on endothelial cells. PlGF has been demonstrated to act synergistically with VEGF and its low expression significantly stimulates the action of VEGF (36).
Tumor necrosis factor-alpha (TNF-a)	TNF-α is a family of pro-inflammatory cytokines with pleiotropic effects. They participate in the pathogenesis of many diseases such as cancer and autoimmune diseases (37). In the case of the eyeball, TNF-a participates in the neovascularization of the retina after hypoxia (38).
Platelet-derived growth factor (PDGF)	PDGF is a cytokine that plays major role in wound healing. Its expression has been confirmed in the cases of many types of cells, such as endothelial cells, macrophages and fibroblasts (39). High expression of this factor has been demonstrated in the case of all retinal proliferative diseases (40). The expression of this factor in the eyeball is probably stimulated by retinal hypoxia. Apart from the proangiogenic effect of PDGF, this cytokine is also a mitogenic and chemotactic factor for RPE and glial cells (41).
Transforming growth factors-b (TGF-b)	TGF-b is a family of cytokines that exhibit pleiotropic effects on tissues. These cytokines are involved in the control of the proliferation and migration of endothelial cells (34, 42). Within the ERM, this cytokine is responsible for the differentiation of cells into fibroblast-like cells – responsible mainly for the production of ERM-building proteins (43).
Angiopoietins	It is a group of proangiogenic cytokines that are of greatest importance in embryonic angiogenesis. It is of great importance in the maturation and stabilization of blood vessels (44, 45).
Interleukin-6 (IL-6)	It is a pleiotropic cytokine that plays an important role in the activation of lymphocytes (46). Increased expression of this cytokine has been observed in the course of retinal injuries and in the case of diabetic patients (47, 48).
Vascular cell adhesion molecules	Intracellular adhesion molecule 1 (ICAM-1), vascular cell adhesion molecule 1 (VCAM-1), E-selectin, and P-selectin play an important role in the circulation of leukocytes in areas of inflammation. TNF-α (and other cytokines) increase their expression, which in turn increases the migration of leukocytes to the surrounding tissues (49, 50).
Tenascin-C	Tenascin-C is an extracellular glycoprotein that modulates cell growth and adhesion. Its participation is essential in the process of blood vessel sprouting (51, 52).
Basic fibroblast growth factor (bFGF)	bFGF is a growth factor and a signaling protein involved in cell growth, morphogenesis and tissue regeneration. It plays an important role in the survival of cells subjected to stress (53). Expression of bFGF and its receptors has been demonstrated within the retina, where they probably influence the course of retinal ischemia (54–56).
Hepatocyte growth factor (HGF)	HGF is a kinase that plays an important role in regulating cell growth and morphogenesis. It has been shown to be pro-angiogenic (57).
Glial cell line-derived neurotrophic factor (GDNF)	GDNF is a cytokine that promotes the survival of many types of nerve cells. It is usually secreted by astrocytes, oligodendrocytes, Schwann cells and motor neurons (58). It has a chemotactic effect on glial cells (59).

## 4. Epidemiology

The main risk factors for ERM are age and PVD. PVD is observed in the case of 70% of patients in the initial stages of the disease. Although PVD can appear early in life or childhood, ERM usually appears after the age of 50, and the risk of its appearance increases exponentially with age.

Gender was not observed to exert any influence as to the risk of the appearance of ERM, although some studies indicate a slight predominance of women among the afflicted (60). Statistically, significant differences in risk of occurrence seem to emerge due to ethnicity – in studies concerning the American society, the highest number of ERM cases is identified in the population of Chinese origin, followed by the ethnic groups of, respectively, Latin American, Caucasian and African descent.

Geographic differences were also observed to play a part: ERM is more common in Europe and South America, and the least common in Asia. This phenomenon is probably related to lifestyle and diet. Other factors contributing to the development of ERM are obesity, type II diabetes, hypertension and hypercholesterolaemia (60, 61).

## 5. Symptoms and diagnosis

The symptoms reported by patients depend on the stage (phase of development) and type of ERM. In some cases, the presence of ERM does not produce clinical symptoms and is diagnosed accidentally. Patients usually complain of: visual disturbances – metamorphopsia, micropsia or macropsia, photopsia, decreased visual acuity, diplopia, and a loss of central vision. The diagnosis of ERM is based on a clinical examination and Optical Coherence Tomography (OCT). In the case of fundoscopy, a cellophane reflex or wrinkling on the retinal surface resulting from the contracture of the membrane can be observed. It usually affects the foveal and parafoveal area. Cystoid macular oedema (CMO), lamellar or full-thickness macular holes (MHs) and/or small retinal hemorrhages can be seen in association with ERM. The diagnosis of idiopathic ERM is based on the exclusion of other ophthalmic diseases like retinal vascular diseases including diabetic retinopathy, retinal vein occlusion, uveitis and other inflammatory diseases, trauma, intraocular tumors, and retinal tear or detachment (62).

**Figure 4:**
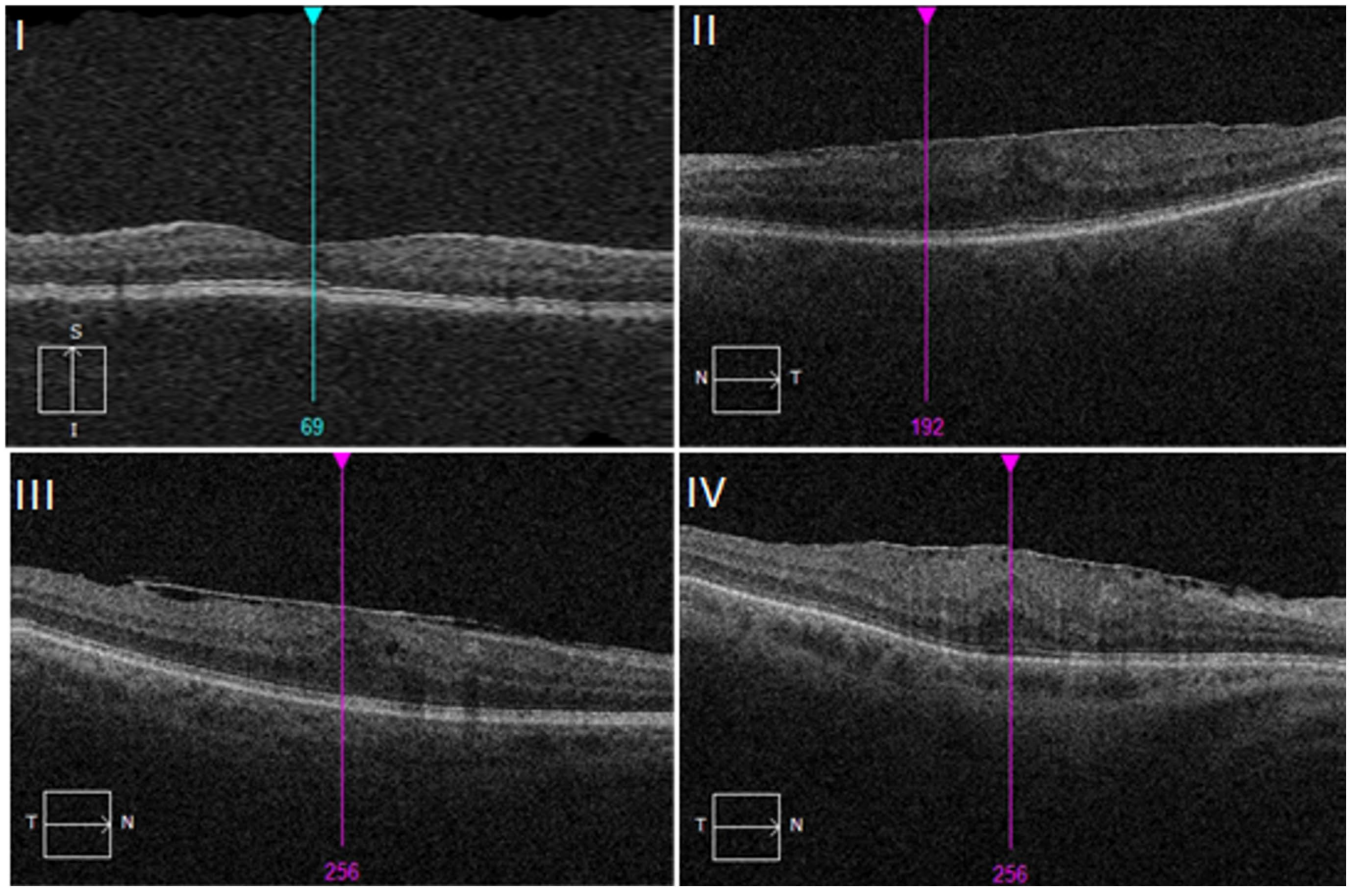
Stages of ERM showed on optical coherence tomography (OCT) images. I – ERMs are mild and thin. Foveal depression is present. II – ERMs with a widening of the outer nuclear layer and loss of the foveal depression. III – ERMs with continuous ectopic inner foveal layers crossing the entire foveal area. IV – ERMs are thick with continuous ectopic inner foveal layers and disrupted retinal layers. Based on clasification by Govetto et al. (64).

There are several ERM classification systems based on OCT findings. No classification, however, is currently suggested for general use in clinical practice (63) (Figure 4).

**Figure 5:**
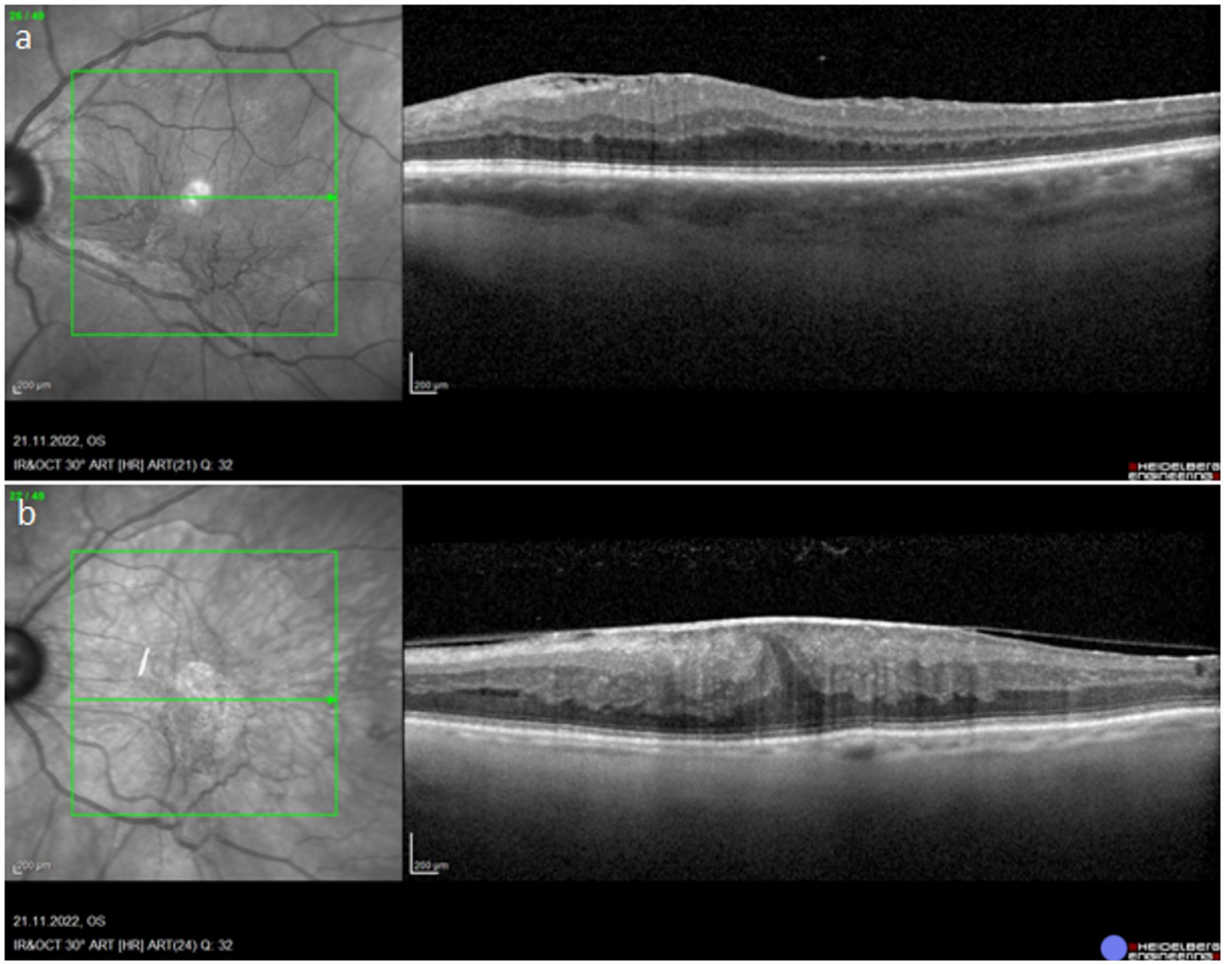
OCT images showing stage 3 and stage 4 of ERM. a – stage 3, b – stage 4.

From the additional diagnostic tools, one worth mentioning is fluorescein angiography (FA), which is useful in the case of secondary ERM when it comes to identifying preoperatively the underlying cause of like intraocular tumors or retinal vascular diseases. Macular edema can also be confirmed with angiography (62). The OCT examination allows not only to deepen the diagnosis, but also to distinguish different stages of the ERM development (64) (Table 4 and Figure 5).

**Table 4:** Optical coherence tomography staging scheme proposed by Govetto et al. (64).

Stage	Description
I	Thin layer of ERM on the retinal surface; foveal depression is present; the layered structure of the retina is preserved
II	Presence of ERM accompanied by the loss of the foveal depression; widening of the retinal outer nuclear layer, the layered structure of the retina is preserved
III	ERMs with continuous (ectopic inner foveal layers) hyporeflective or hyperreflective band, extending from the inner nuclear layer (INL) and inner plexiform layer (IPL) crossing the entire foveal area; foveal depression is absent; the layered structure of the retina is preserved
IV	Thick ERM with continuous ectopic inner foveal layers; foveal depression is absent; distortion of the retinal layers

## 6. ERM treatment

The management options for ERM are limited and consist of observation or surgical intervention. Official guidelines for performing the surgical ERM removal have not been established. Before taking appropriate intervention measures, it is advisable to discuss with the patient all the possible benefits and complications related to the surgery in relation to the severity of the patient’s symptoms and their lifestyle (65).

During the surgical intervention called pars plana vitrectomy (PPV), the epiretinal membrane is removed and the retinal tractions are released. ILM is considered to be the scaffold for myofibroblast proliferation, thus it is commonly removed alongside with ERM, in order to minimize the risk of ERM recurrence. Sometimes PPV is combined with a simultaneous cataract surgery involving intraocular lens implantation (phacovitrectomy). During the surgery, special dyes, like triamcinolone acetonide, trypan blue, indocyanine green (ICG), and brilliant blue, are used to distinguish ERM from the retinal layers (66).

It is possible to discern two types of PPV – complete vitrectomy, which involves the whole vitreous body being detached from the retina and removed, and limited vitrectomy, which involves removing only the central part of the vitreous body. Most of the times, the complete vitrectomy is performed, however, there is no difference when it comes to the results of it and the limited vitrectomy. Limited vitrectomy is usually faster and potentially produces fewer long-term side effects (67).

The surgical treatment of ERM provides excellent postoperative visual outcomes and is a relatively safe procedure. Improvement in the vascularity of the choroid was also observed (68). Like any other surgical intervention, ERM surgery can cause complications, such as endophtalmitis, retinal detachment or ERM recurrence (69).

## 7. Conclusion

It is estimated that the main risk factor that significantly preceding the development of ERM, i.e., PDV, occurs in about 2% of the population. The main epidemiological factor associated with PDV is old age (60, 61, 70). An increased incidence of ERM is therefore to be expected due to the aging of the population. By understanding the successive processes leading to the formation of ERM, we better understand the causes of not only this pathology but also PDV. Currently, it is not possible to predict the development of this pathology based on biochemical tests, while the development of knowledge about the histological structure of ERM and the expression of cytokines and molecules related to ERM pathophysiology may in the future allow for the detection of a biochemical marker allowing for early detection of ERM development without the need for costly OCT, development guidelines for qualifying patients for surgical treatment and a potential conservative treatment regimen. Currently, there is also no prophylaxis for idiopathic ERM development, but by understanding the inflammatory factors involved in this process, it will be possible to develop some. In the case of secondary ERM, in addition to reducing the risk by appropriate treatment of the cause, it is possible, thanks to potential biochemical markers, to include some of these patients in regular observation due to high risk of ERM development.

## Author contributions

MO and MN-W: conceptualization and writing—original draft preparation. WR: writing—review and editing, and supervision. All authors contributed to the article and approved the submitted version.

## Conflict of interest

The authors declare that the research was conducted in the absence of any commercial or financial relationships that could be construed as a potential conflict of interest.

## Publisher’s note

All claims expressed in this article are solely those of the authors and do not necessarily represent those of their affiliated organizations, or those of the publisher, the editors and the reviewers. Any product that may be evaluated in this article, or claim that may be made by its manufacturer, is not guaranteed or endorsed by the publisher.
